# Metabolomics analysis reveals novel serum metabolite alterations in cancer cachexia

**DOI:** 10.3389/fonc.2024.1286896

**Published:** 2024-02-20

**Authors:** Tushar H. More, Karsten Hiller, Martin Seifert, Thomas Illig, Rudi Schmidt, Raphael Gronauer, Thomas von Hahn, Hauke Weilert, Axel Stang

**Affiliations:** ^1^ Department of Bioinformatics and Biochemistry, Braunschweig Integrated Centre of Systems Biology (BRICS), Technische Universität Braunschweig, Braunschweig, Germany; ^2^ Asklepios Precision Medicine, Asklepios Hospitals GmbH & Co KgaA, Königstein (Taunus), Germany; ^3^ Connexome GmbH, Fischen, Germany; ^4^ Department of Human Genetics, Hannover Medical School, Hannover, Germany; ^5^ Hannover Unified Biobank (HUB), Hannover, Germany; ^6^ Immunetrue, Cologne, Germany; ^7^ Asklepios Hospital Barmbek, Department of Gastroenterology, Hepatology and Endoscopy, Hamburg, Germany; ^8^ Asklepios Tumorzentrum Hamburg, Hamburg, Germany; ^9^ Semmelweis University, Asklepios Campus Hamburg, Budapest, Hungary; ^10^ Asklepios Hospital Barmbek, Department of Hematology, Oncology and Palliative Care Medicine, Hamburg, Germany

**Keywords:** cancer cachexia, GC-MS metabolomics, erythronic acid, glucuronic acid, serum metabolites, metabolic pathways, body metabolism

## Abstract

**Background:**

Cachexia is a body wasting syndrome that significantly affects well-being and prognosis of cancer patients, without effective treatment. Serum metabolites take part in pathophysiological processes of cancer cachexia, but apart from altered levels of select serum metabolites, little is known on the global changes of the overall serum metabolome, which represents a functional readout of the whole-body metabolic state. Here, we aimed to comprehensively characterize serum metabolite alterations and analyze associated pathways in cachectic cancer patients to gain new insights that could help instruct strategies for novel interventions of greater clinical benefit.

**Methods:**

Serum was sampled from 120 metastatic cancer patients (stage UICC IV). Patients were grouped as cachectic or non-cachectic according to the criteria for cancer cachexia agreed upon international consensus (main criterium: weight loss adjusted to body mass index). Samples were pooled by cachexia phenotype and assayed using non-targeted gas chromatography-mass spectrometry (GC-MS). Normalized metabolite levels were compared using *t*-test (p < 0.05, adjusted for false discovery rate) and partial least squares discriminant analysis (PLS-DA). Machine-learning models were applied to identify metabolite signatures for separating cachexia states. Significant metabolites underwent MetaboAnalyst 5.0 pathway analysis.

**Results:**

Comparative analyses included 78 cachectic and 42 non-cachectic patients. Cachectic patients exhibited 19 annotable, significantly elevated (including glucose and fructose) or decreased (mostly amino acids) metabolites associating with aminoacyl-tRNA, glutathione and amino acid metabolism pathways. PLS-DA showed distinct clusters (accuracy: 85.6%), and machine-learning models identified metabolic signatures for separating cachectic states (accuracy: 83.2%; area under ROC: 88.0%). We newly identified altered blood levels of erythronic acid and glucuronic acid in human cancer cachexia, potentially linked to pentose-phosphate and detoxification pathways.

**Conclusion:**

We found both known and yet unknown serum metabolite and metabolic pathway alterations in cachectic cancer patients that collectively support a whole-body metabolic state with impaired detoxification capability, altered glucose and fructose metabolism, and substrate supply for increased and/or distinct metabolic needs of cachexia-associated tumors. These findings together imply vulnerabilities, dependencies and targets for novel interventions that have potential to make a significant impact on future research in an important field of cancer patient care.

## Introduction

Cancer cachexia is a common adverse effect of cancer with negative impact on patient´s physical function, quality of life, and survival (1). Involuntary weight loss (WL), adjusted to the body mass index (BMI), represents the validated cardinal criterium of the international consensus-definition for cancer cachexia, which distinguishes between cachectic and non-cachectic patients with regards to all other cachexia domains proposed (e.g., C-reactive protein [CRP], food intake, appetite loss, performance status [PS]) (1–3). Cachexia is estimated to affect 50-80% of cancer patients, which worsens the susceptibly to toxic side effects of anti-cancer drugs, and to account for up to 20% of cancer deaths (4, 5). Despite its clinical relevance, this cancer-associated body wasting syndrome remains without effective treatment. Current concepts outline a tumor-orchestrated takeover of the whole-body metabolism to promote tumor anabolism and growth at the expense of host tissue catabolism (6, 7). Serum metabolites take part in pathophysiological processes of CC, but apart from altered levels of select metabolites, there is relative paucity of data on the global alterations of the serum metabolome, which integrates the functional readout of the whole-body metabolic state (8–10). Determining variations of the serum metabolome may reveal yet unknown metabolite alterations, broaden the scope of dysregulated metabolic pathways, and drive the translation of chemical metabolome data into biological knowledge (11–13). These results, in turn, may instruct strategies for novel therapeutic interventions of greater clinical benefit.

Metabolomics, or comprehensive metabolite profiling, uses analytical chemistry platforms, such as mass spectroscopy coupled with gas chromatography (GC-MS), to provide an integrated status of the metabolome to metabolic disease research (10, 14). Serum metabolomics, in non-targeted mode, has proven utility for discovering unanticipated metabolites and new metabolic pathways that change between clinical states, and hence for the design of novel intervention strategies aimed at modulating metabolic diseases (10, 13–15). Since metabolites interact and the structures of metabolomics data are complex, significant metabolites are not necessarily good predictors (15, 16). Therefore, consistent results from both statistical (direct metabolite-level testing between sample groups) and machine learning (ML) methods (train models to label groups of samples) lend strengths to metabolomics study findings, as these complementary methods differentially process the data and validate each other (16–18).

Here, we comprehensively characterize the serum metabolite profiles of 78 cachectic and 42 non-cachectic cancer patients using a non-targeted metabolomics approach followed by pathway analysis. Our objectives were to assess statistically differential cachexia-related serum metabolite alterations and metabolome clusters, to examine top differential metabolite features for associated pathways to help understand what these metabolite changes represent, and to apply an ML strategy to evaluate whether significance-based and prediction-oriented results are distinct or overlapping. Altogether, we aimed to provide a resource for future research that can help define testable hypotheses about mechanisms of action and/or design approaches for novel therapeutic strategies in an important field of cancer patient care.

## Materials and methods

### Study population

For this cross-sectional case-control study, adult patients with newly diagnosed, metastatic cancer (stage UICC IV) were recruited via the cancer care in- and out-patient units and oncology wards of the Asklepios Hospital Barmbek between January 2019 and December 2021. Patients were included at the time of diagnosis before start of any anticancer treatment. Primary cancer types included gastric, colorectal, pancreatic, liver and ovarian cancer. Eligible patients met the following inclusion criteria: ≥18 years of age, and histologically proven metastatic cancer diagnosis, and either antibiotics treatment within ≤2 weeks or non-exposure of ≥3 months before inclusion. Exclusion criteria were as follows: acute or chronic diarrhea, acute gastrointestinal illness including ileus, inflammatory bowel disease, acute infection, autoimmune diseases, immunosuppressive therapy including corticosteroids, acquired immunodeficiency syndrome, kidney or liver failure, and need for emergency surgery. and. The study was approved by the Ärztekammer Hamburg, Protocol number: V5649, Date: 23.10.2017 and was conducted according to the Declaration of Helsinki and its revisions. All patients gave written informed consent.

### Clinical assessments

Demographic information (age, gender, cancer type, medication, CRP values) was collected from medical records by the study coordinators at the time of study inclusion. Patient-reported data on height, body weight, WL history, appetite, food intake, and vegetarian diet were collected by means of a structured questionnaire. A research assistant was available and provided help in a face-to-face interview as necessary. Information about actual height and weight, WL at last 6 months, and food intake past month (unchanged or reduced) was provided by the patients using questions from the Scored Patient-Generated Subjective Global Assessment (PG-SGA) (19). Assessment of appetite was performed using a numerical rating scale provided by the Edmonton Symptom Assessment System, scoring 0 (normal appetite) to 10 (no appetite) (20). Diet-based vegetarianism was determined from the intake of animal products (red meat, poultry, fish, dairy products, and eggs). Vegetarians were defined by a plant-based dietary pattern that excludes red meat, and, to different extents, other animal products (subtypes included lacto-/, ovo-/, pesco-/, lacto-ovo-/and pesco-lacto-ovo-vegetarians and vegans depending on the inclusion and exclusion of poultry, fish, dairy products and/or eggs). Smoking was defined as current daily smoking. We used self-reported average absolute alcohol consumption (grams per week) during the last 12 months. Medication use was defined as a drug purchase during the 3 months preceding the study inclusion. Prevalent diabetes was defined as self-reported diabetes, a diabetes diagnosis code indicating diabetes in medical records, and/or use of diabetes medications. Patients were classified into two groups (cachexia versus non-cachexia) based on the agreed and validated diagnostic criteria from the international consensus (1–3). The criterion for cachexia was: WL ≥5% the past 6 months or WL ≥2% the last 6 months and BMI ≤20 kg/m^2^ (1). Patients above or below theses cut-offs were grouped as cachexia or non-cachexia. BMI is reported as current weight (kg)/height (m)^2^.

### Sample collection

Morning overnight fasting (≥6 hours) venous blood (5-10 mL) was collected in serum tubes. The blood samples were kept at 4°C for 20 minutes for clotting. Clotted samples were centrifuged at 1300 x g for 10 minutes at 4°C. The removed supernatant serum samples were immediately stored at -80°C within ≤30-40 minutes after blood collection. Samples remained stored on average for 1.2 ± 0.6 years until further processing. Finally, the samples were transported to the Department of Bioinformatics and Biochemistry, Braunschweig Integrated Centre of Systems Biology (BRICS), University of Braunschweig, on dry ice for metabolomics analysis.

### Metabolite extraction

Metabolite extraction was performed as per our previous publication (21). Prior to extraction, the serum samples were thawed on ice for 30 minutes. Then, 11 µL of serum was mixed with 100 µL of an extraction solvent consisting of methanol and water (at a ratio of 8:1) at -20°C. This solvent also contained internal standards, specifically 2 µg/mL of D6-glutaric acid and U13C-ribitol. The mixture was vortexed for 10 minutes at 1400 rpm and 4°C, followed by centrifugation at 13,000 g and 4°C for 10 minutes to precipitate proteins. The resulting supernatants (90 µL) were transferred to glass vials and evaporated using speed-vac at 4°C. The metabolic extracts in the glass vials were sealed with a crimped aluminium cap featuring a septum to prevent oxidation. Typically, the samples were extracted and analyzed immediately after extraction. If storage was necessary, the dried samples were stored at -20°C until GC-MS measurement. The storage time until analysis did not exceed 48 hours. Serum samples were individually extracted in technical triplicates. A fraction (10 µL) from each sample is used to create a pooled quality control (QC) sample, which is then extracted and acquired after every 8th measurement. These QC samples served as a means to normalize untargeted metabolomics data by dividing the sample metabolite intensity by the average intensity of the nearest pool sample, ensuring measurement quality (22).

### Metabolic analysis

Metabolomics measurements were performed using gas chromatography coupled with mass spectrometry (GC-MS). To render the identification of polar metabolites, two-step derivatization was performed prior to analysis. Metabolite extracts were derivatized using a multipurpose sampler (Gerstel MPS). The first derivatization was performed by adding 15 µL of (20 mg/mL) methoxyamine hydrochloride in pyridine (Sigma-Aldrich), shaken for 90 min at 40°C. The second derivatization was performed by adding an equal volume of N-methyl-N-trimethylsilyl-trifluoroacetamide (MSTFA) (Macherey-Nagel) under continuous shaking for 30 min at 40°C. The sample (1 µL) was injected into an SSL injector at 270°C in spitless mode.

GC-MS measurements were performed on Agilent 7890A GC equipped with a 30 m DB-35MS + 5m Duraguard capillary column (0.25 mm inner diameter, 0.25 µm film thickness), which was connected to an Agilent 5977B MSD. Helium was used as the carrier gas at a flow rate of 1.0 mL/min. The GC oven temperature was held at 80°C for 6 min, subsequently increased to 300°C at 6°C/min, and held at that temperature for 10 min. The temperature was increased to 325°C at 10°C/min and held for an additional 4 min, resulting in a total run time of 60 min per sample. The transfer line temperature was set to 280°C, and the MSD was operating under electron ionization at 70 eV. The MS source was held at 230°C and the quadrupole at 150°C. Full scan mass spectra were acquired from m/z 70 to m/z 800 at a scan rate of 5.2 scans/s. Pooled samples were measured after every eighth GC-MS measurement for quality control and data correction.

### Data processing

All GC-MS chromatograms were processed using our in-house software (23). The software package supports the deconvolution of mass spectra, peak picking, integration, and retention index calibration. Compounds were identified using an in-house mass spectral library by spectral and retention index similarity. The following deconvolution settings were applied to scan data: peak threshold: 5; minimum peak height: 5; bins per scan: 10; deconvolution width: 7; no baseline adjustment; minimum 15 peaks per spectrum; no minimum required base peak intensity. Retention index calibration was based on a C10–C40 even n-alkane mixture (68281, Sigma-Aldrich, Munich, Germany). Relative quantification was done using the batch quantification function of our in-house software (23). Data were normalized to quality control pool measurement and intensity of the internal standard (D6-Glutaric acid).

### Statistical analysis

Group-wise comparisons of cachectic versus non-cachectic cancer patients with regards to items representing cachexia domains and a range of clinical data were performed. Continuous variables with normal distribution are presented as mean (standard deviation) and Welch`s two sample t-test was used to examine differences between groups. Continuous variables outside the normal distribution are presented as medians (quartile 1, quartile 3) and Wilcoxon rank sum test was applied to examine differences between groups. Categorical variables were summarized as counts (percentages) and Pearson`s Chi-squared test was applied to test for differences between groups. Different statistical approaches were utilized to identify significant metabolic differences between the cachexia and non-cachexia groups. At first, metabolomics data were cube root transformed and range scaled to obtain Gaussian distribution using Metaboanalyst 5.0 (24). Principal component analysis (PCA) was applied to detect intrinsic clustering, while partial least squares discriminant analysis (PLS-DA) was used for supervised clustering. Cross-validation was performed to avoid over-fitting, and R2 and Q2 values were employed to evaluate the model’s goodness of fit and predictive ability. The variable importance in projection (VIP) score was utilized to extract variables that significantly influenced group discrimination, with a VIP score greater than 1 considered important. Additionally, significant metabolic differences were confirmed within the cachexia and non-cachexia groups using a t-test (p < 0.05) that was adjusted for multiple hypothesis testing using false discovery rate (FDR) correction. The collective analysis was used to determine significant metabolic differences in the cachexia versus the non-cachexia group. In addition, the volcano plot was used to visualize the alterations in metabolites between cachectic and non-cachectic cancer patients. Box-and-whisker plots were generated using SPSS (V27), and heat maps of altered metabolites were created using MetaboAnaylst 5.0. The pathway analysis tool (MetPA) in MetaboAnalyst 5.0 was utilized for the pathway analysis of significant metabolites (24).

### Machine learning classification

For all ML-based approaches, we used the Waikato Environment for Knowledge Analysis (Weka) (https://www.cs.waikato.ac.nz/ml/weka/) (25). For developing a predictive ML model for binominal classification between cachectic and non-cachectic patients to predict the cachexia state, we applied simple logistic regression analyses, because they are typically applied and useful to investigate biomedical regression and classification issues. Simple Logistics in Weka fits a multinomial logistic regression model using the LogitBoost algorithm (26). The number of LogitBoost iterations was manually selected based on an optimization of cross-validation results. We applied a meta classifier approach with reweighted training instances to make base predictors cost-sensitive for balancing positive and negative predictive values, predominantly to avoid false positive prediction and to improve overall true predictive accuracy. Further, we applied a 10-fold cross-validation, with each fold containing a balanced proportion of compared groups to handle dataset imbalances and to avoid overfitting. After applying the trained Simple Logistics model to classify the left-out test set, model´s classification performance was estimated by receiver operating characteristic (ROC) methods and by coefficient analysis to determine the predictor composition and predictors contributing to ML models predictive performance.

## Results

### Clinical characteristics of the study population

**Figure 1:**
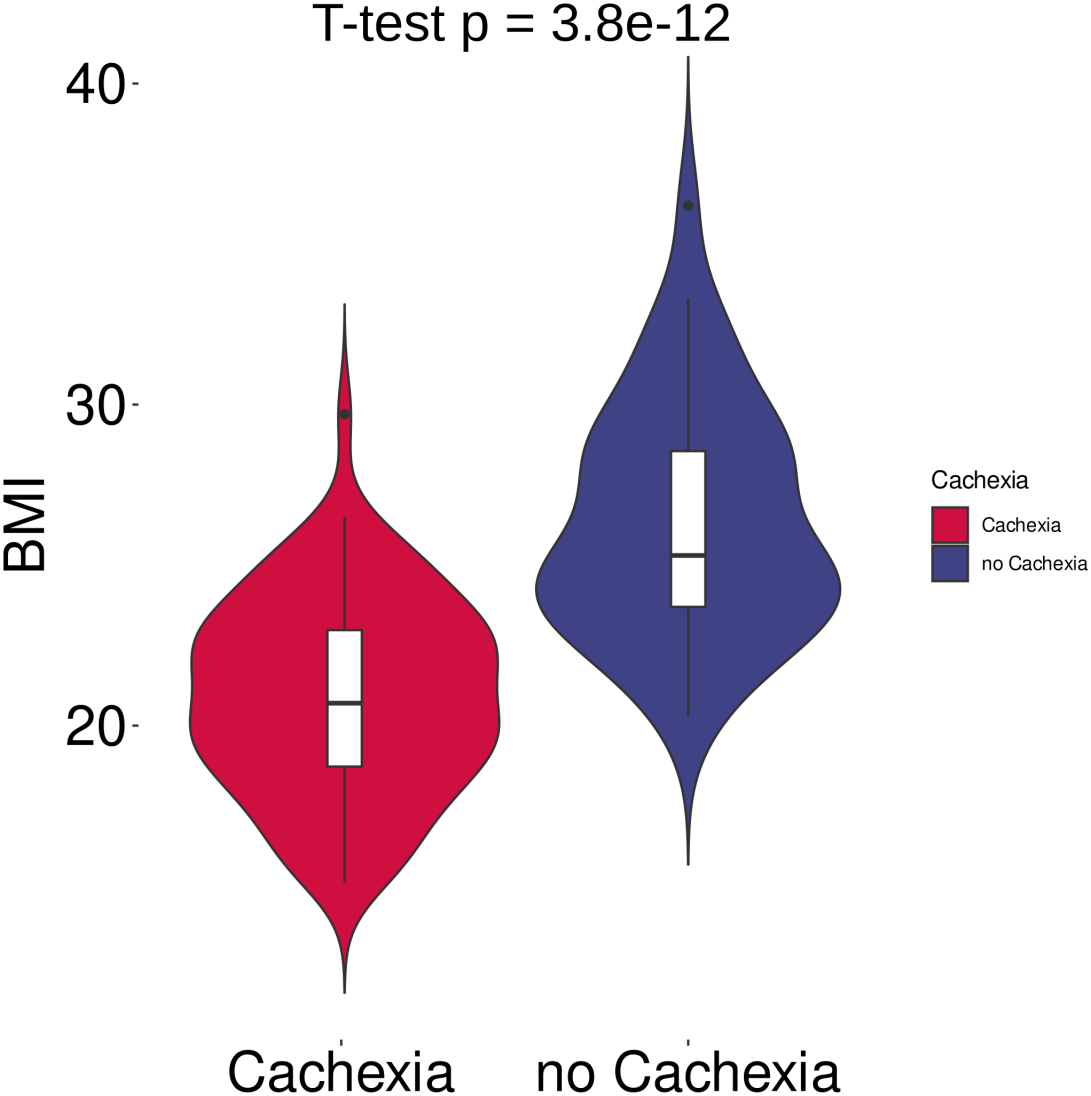
Violin plots of body mass index (BMI) in cachectic (n = 42) and non-cachectic (n = 78) patients. BMI differed significantly between cachectic and non-cachectic cancer patients (*p* < 0.001, two-tailed unpaired *t*-test).

In total, 120 cancer patients with metastatic disease (UICC stage IV) participated in the study. 41 patients were diagnosed with colorectal cancer, 32 patients with pancreatic cancer, 30 with gastric cancer, 12 with liver cancer, and 5 with ovarian cancer. Among these, 78 patients were classified as cachectic, while 42 patients were non-cachectic (**Supplementary Table 1**). The cachectic patients had a mean BMI of 20.9 kg/m^2^ and a mean WL of 6.5 kg (mean %WL: -9.7%), while the non-cachectic patients had a mean BMI of 26.4 kg/m^2^ without WL (**Figure 1**, **Supplementary Table 1**, *p* < 0.001, respectively). When comparing cachectic versus non-cachectic patients on items representative of key cachexia domains, higher levels of CRP (median 37 versus 14 mg/dl, *p* < 0.001) and appetite loss (median score 4.0 versus 2.0, *p* < 0.001) and reduced food intake (76% versus 26%, *p* < 0.001) was observed for cachectic patients. The ECOG-PS was significantly lower in cachectic patients compared to non-cachectic patients (ECOG ≥2: 44% versus 12%, *p* < 0.001). There were no significant differences in the distribution of cancer types between the two groups analyzed (**Table 1**, *p* > 0.05, respectively). Moreover, the two groups matched in clinical factors (e.g., sex, age, alcohol intake, smoking, diabetes, medication) that could potentially affect the serum metabolite profile (**Table 1**, *p* > 0.05). Overall, these data underline the legitimacy of using BMI-adjusted WL as diagnostic criterium for cancer cachexia and that there was almost equal distribution of covariables without significant difference between the cachectic and non-cachetic patient group.

**Table 1:** Demographic, clinical and cachexia domain characteristics of the study population (n = 120 cancer patients with metastatic disease, stage UICC IV).

Cohort	Cachexia	No Cachexia	P-value
Sample size (n)	78	42	
Patient data			
Age,^1^ *year*	68 (10)	65 (12)	0.074
Male^3^	46 (59)	17 (40)	0.053
ECOG-PS,^3^ s*core* ≤*1*	44 (56)	37 (88)	<0.001
Body mass index (BMI)			
Height,^1^ *cm*	172 (10)	172 (10)	0.880
Weight,^1^ *kg*	62 (12)	78 (13)	<0.001
BMI,^1^ *kg/m^2^*	20.9 (3.0)	26.4 (3.6)	<0.001
Weight loss (WL)			
WL,^1^ *kg*	-6.5 (1.8)	-0.2 (1.6)	<0.001
WL,^1%^	-9.7 (3.6)	-0.3 (2.0)	<0.001
Food intake			
Reduced,^1^ (*vs. unchanged)*	59 (76)	11 (26)	<0.001
Appetite loss			
ESAS Score,^2^ *score 0-10*	4.0 (3.0, 5.0)	2.0 (0.25, 2.0)	<0.001
C-reactive protein (CRP)			
CRP values^2^ (mg/dl)	37 (9, 61)	14 (7, 30)	<0.001
Clinical data			
Smoking^3^	23 (29)	10 (24)	0.682
Alcohol^3^	26 (33)	17 (40)	0.713
Vegetarian^3^	5 (6.4)	2 (4.7)	1.0
Diabetes	17 (21)	7 (17)	0.644
Cancer type			
Colon Cancer^3^	25 (32)	16 (38.1)	0.707
Pancreatic Cancer^3^	23 (29)	9 (21)	0.532
Gastric Cancer^3^	20 (26)	10 (24)	1.0
Liver Cancer^3^	7 (9)	5 (12)	0.754
Ovarian Cancer^3^	3 (3.8)	2 (4.8)	1.0
Cancer stage			
Metastatic Disease (stage UICC IV)^3^	78 (100)	42 (100)	1.0
Medication			
Morphine^3^	20 (25)	7 (17)	0.499
Novaminsulfon^3^	23 (29)	9 (21)	0.523
Non-steroidal Analgetics^3^	14 (18)	10 (24)	0.642
Pantozol^3^	8 (10)	7 (16)	0.403
Diuretics^3^	7 (9)	5 (12)	0.754
Antibiotics (within ≤2 weeks)^3^	20 (25)	10 (24)	1.0
No Antibiotics (within ≥3 months)^3^	58 (75)	32 (76)	1.0

^1^Mean (standard deviation) [normal data distribution]; Welch two sample t-test applied to compare groups.

^2^Median (quartile 1, quartile 3) [outside normal distribution]; Wilcoxon rank sum test applied to compare groups.

^3^Count (percentage); Pearson´s Chi-squared test applied to compare groups.

### Metabolic profiling reveals distinctive patterns between patients with and without cachexia

**Figure 2:**
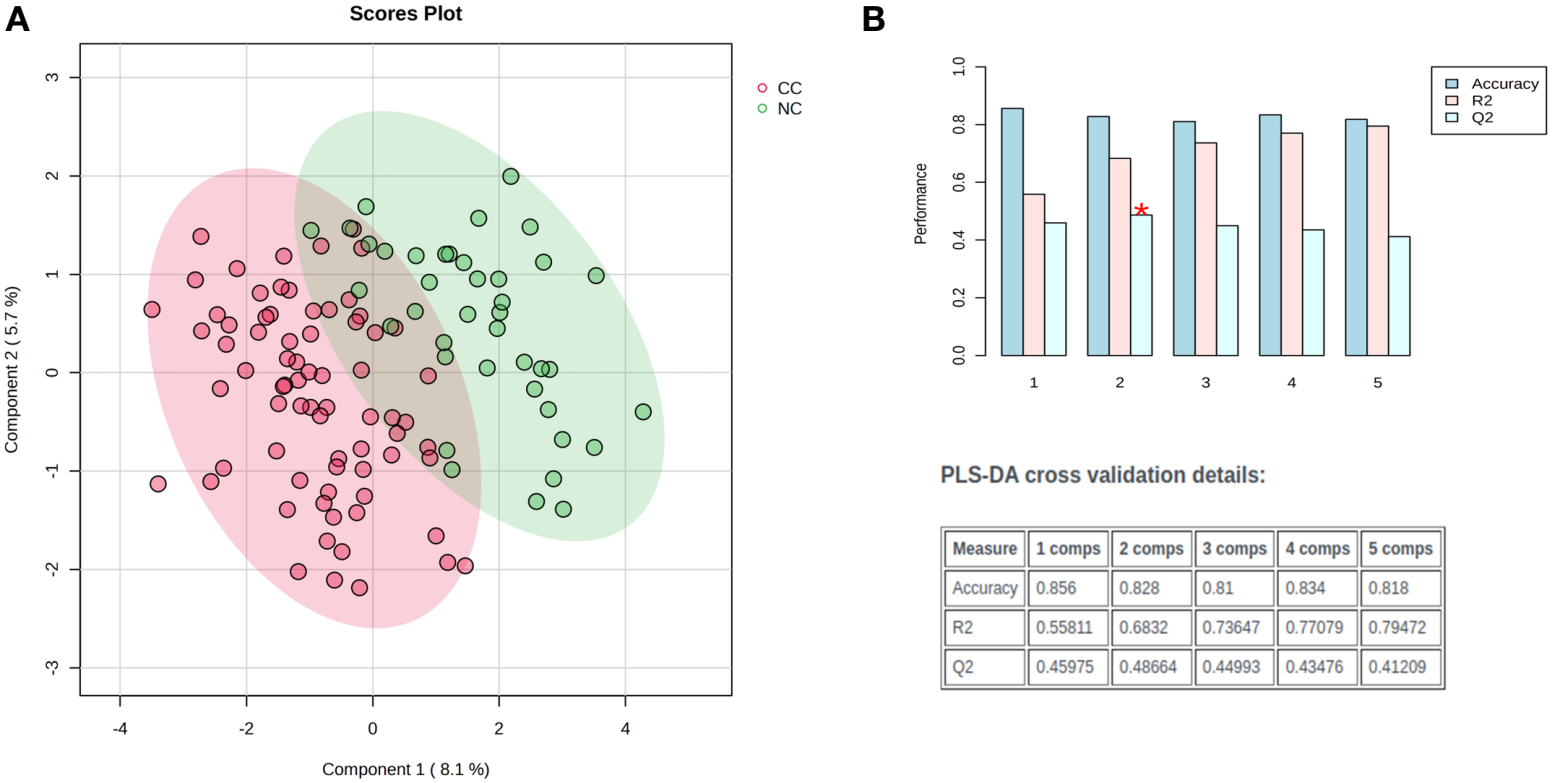
Exploratory multivariate statistical analysis. **(A)** Partial least square discriminant analysis (PLS-DA) score plots distinct clustering of cachectic (red) and non-cachectic (green) cancer patients. **(B)** The PLS-DA model was evaluated for its validity using a random permutation test that involved 100 permutations. The plot generated after the test highlighted the best classifier (a red asterisk) with an R2 value of 0.69, indicating the amount of variance explained by the model, and a Q2 value of 0.48, which indicated its predictive ability. A high R2 and Q2 value indicates good predictive ability and confirms the validity of the PLS-DA model. The accuracy of the best model is summarized in an inset table, which includes Q2, R2, and the number of components used in the model. “Comps” refer to the number of components.

Distinct patterns emerged in cancer patients, distinguishing those with and without cachexia following exploratory statistical analysis. Within the metabolic profiling of serum samples, 159 prevalent metabolites were identified, and leveraging an in-house metabolic reference library facilitated the annotation of 60 metabolites. The normalization process employed pooled quality controls and D6-Glutaric acid peak areas as internal standards. Furthermore, the data matrix underwent log transformation and Pareto scaling to achieve a Gaussian distribution. Principal component analysis discerned inherent clusters and outliers within the metabolic dataset (**Supplementary Figure S1**). Further, distinct clusters between cachexia and non-cachexia groups were evident following partial least square discriminant analysis (PLS-DA). Model validation, executed through 100 randomly permuted models in cross-validation analysis (**Figure 2A**), demonstrated cumulative R2 and Q2 values of 0.69 and 0.48, respectively, emphasizing the robust predictive ability of the original model (**Figure 2B**).

### Significant metabolite differences unveiled between cachectic and non-cachectic patients

**Figure 3:**
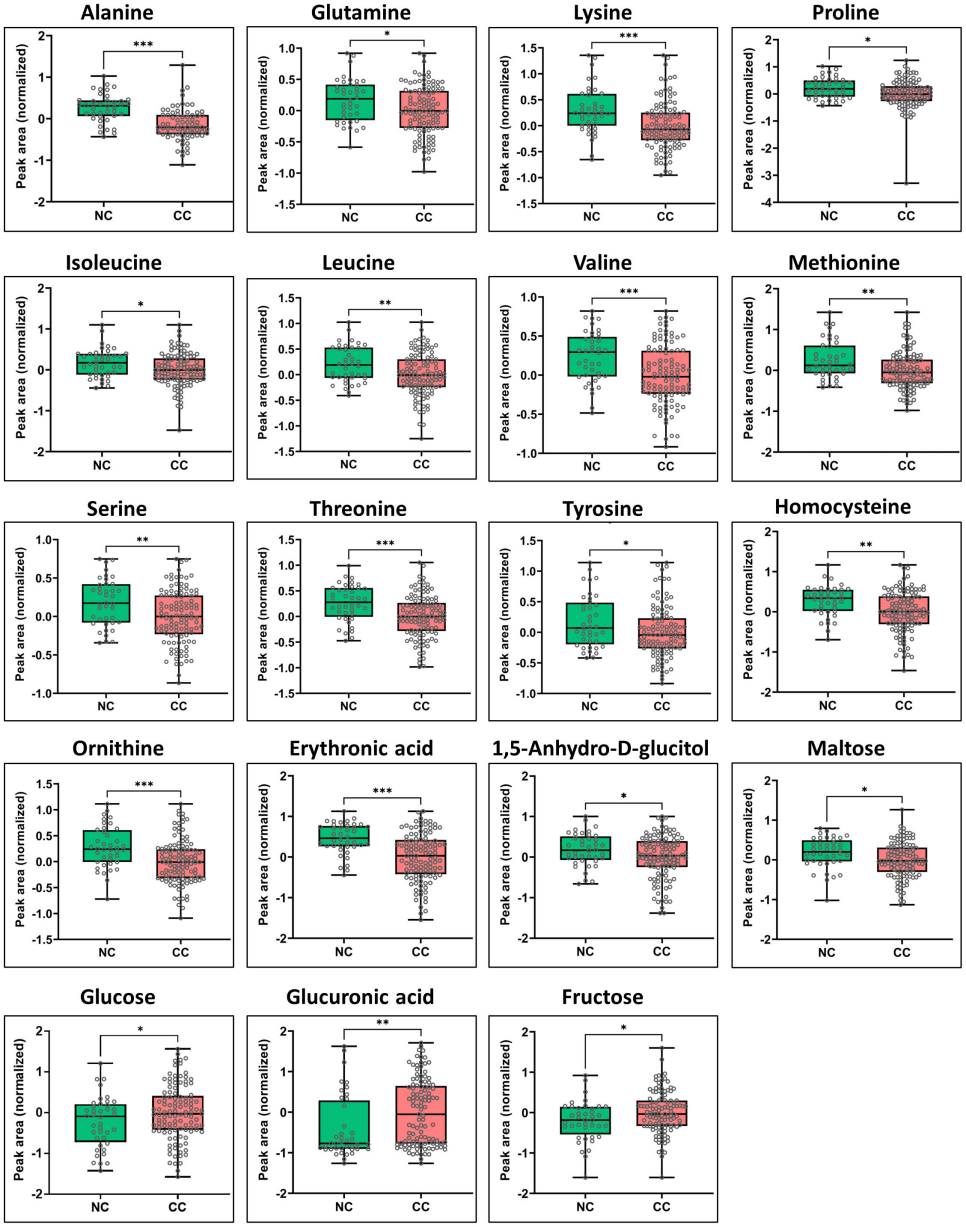
Box-and-whisker and dot plots showing significant differences in serum levels of specific metabolites between cachectic and non-cachectic cancer patients. Specific significant metabolite differences were obtained after Tukey’s HSD and illustrated as normalized peak area differences. *P ≤ 0.05; **P ≤ 0.01; *** P ≤ 0.0001 and all lower values.

**Figure 4:**
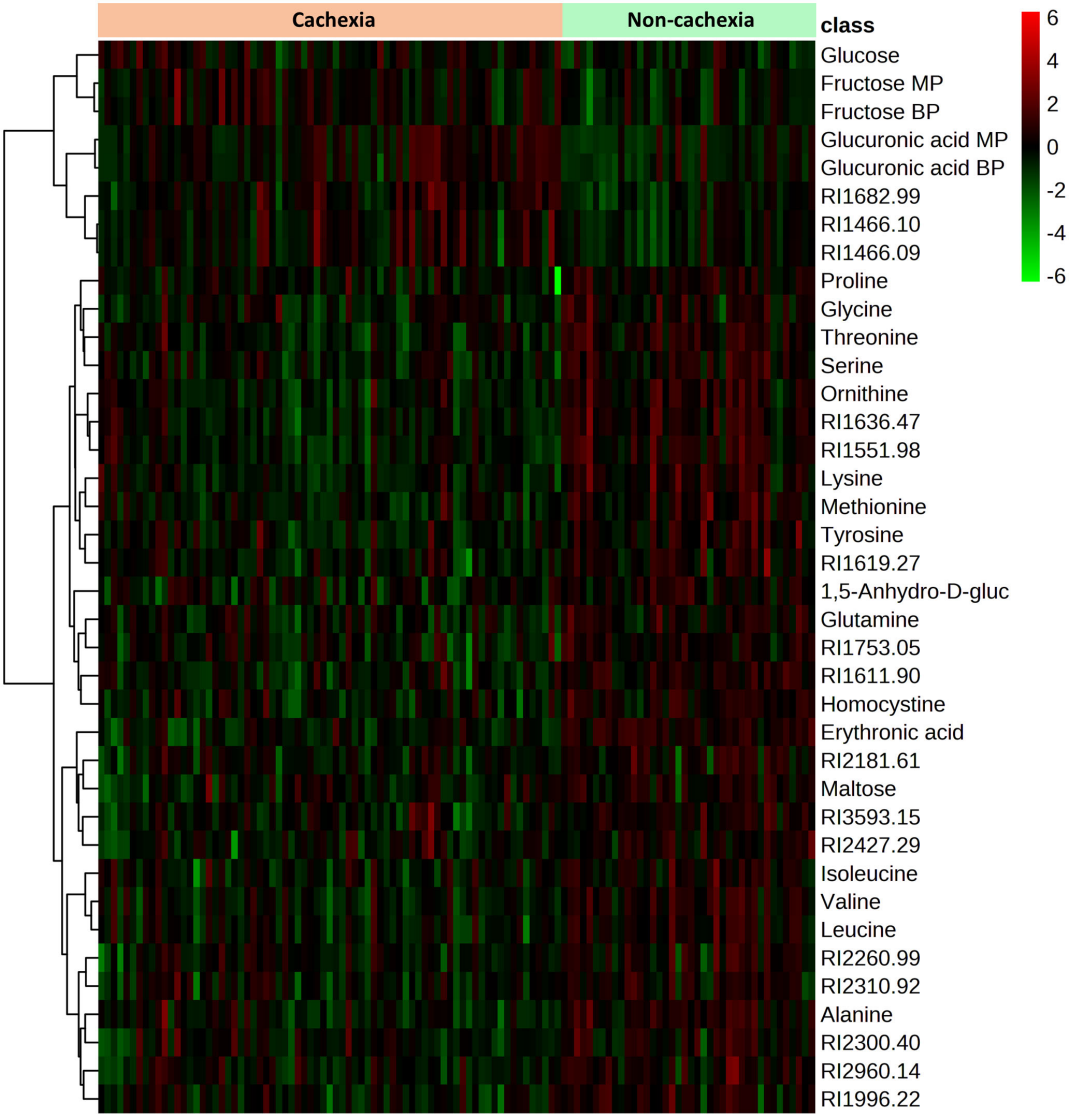
Heatmap showing the 38 metabolites with significant differences in serum level between cachectic and non-cachectic cancer patients. Significant differences were determined by false discovery rate (FDR)-corrected *t*-test p-values (FDR-corrected *p* < 0.05) to adjust for multiple hypothesis testing. The colors from green to red indicate increased metabolite concentration (normalized peak area).

**Figure 5:**
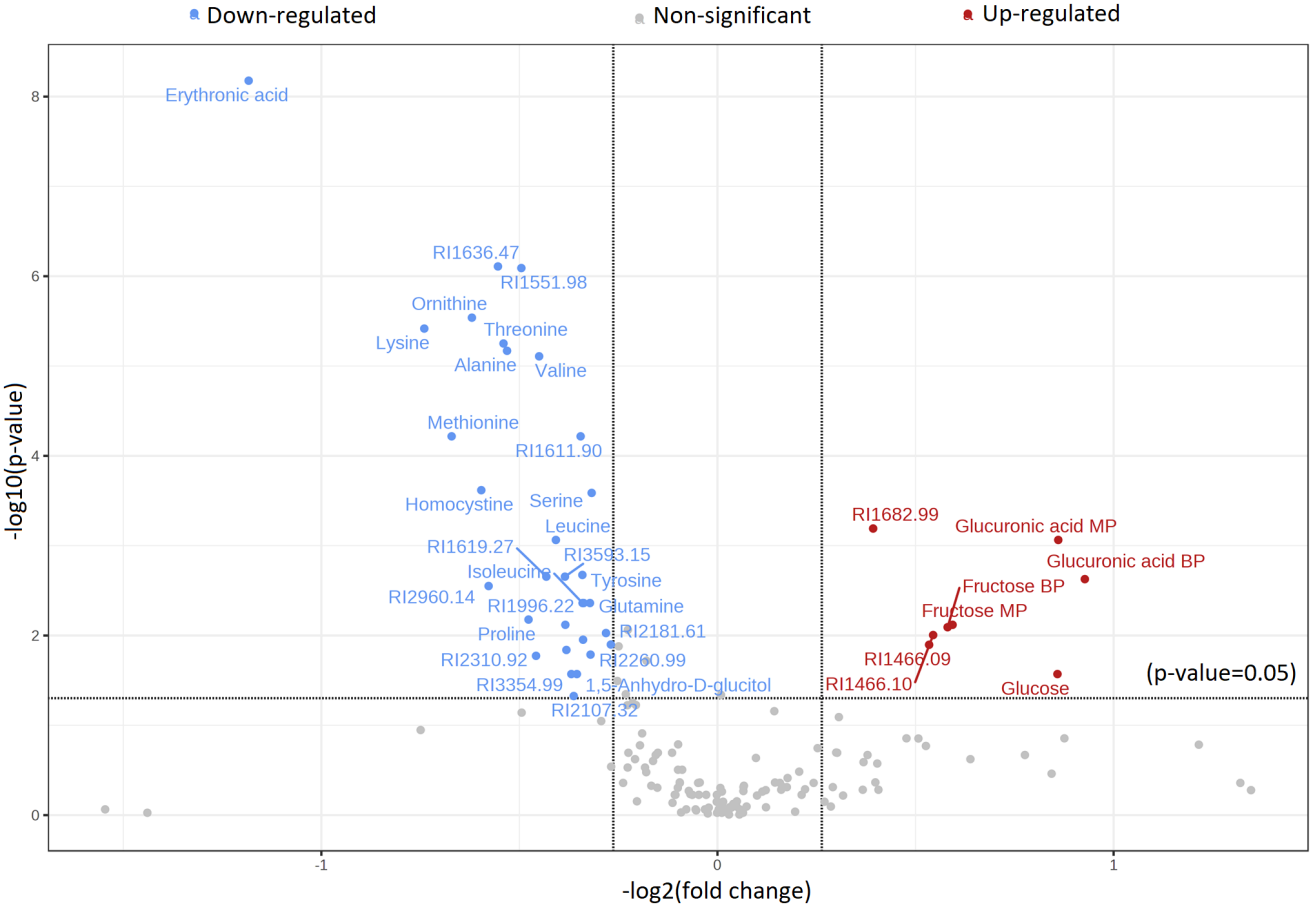
Volcano plot displaying the distribution of significantly altered metabolites with identification in a group comparison between cachectic and non-cachectic cancer patients. Red represents significantly up-regulated metabolites, blue represents significantly down-regulated metabolites, and grey represents metabolites with no difference in comparative analysis between cachectic and non-cachectic cancer patients. Metabolites with a *t*-test p-value less than 0.05 were selected, and the results were adjusted for multiple hypothesis testing using the false discovery rate (FDR).

Significant metabolic differences were revealed through a rigorous analysis of cachexia and non-cachexia groups using various statistical methods. Initially, a *t*-test revealed significant metabolic alterations (FDR-adjusted *p* < 0.05) in both groups. Subsequently, the PLS-DA model’s VIP score identified key variables influencing group discrimination. This comprehensive examination revealed distinctive metabolic differences in 38 metabolites between the cachectic and non-cachectic patients (**Supplementary Table S1**). Among these, 19 metabolites were confidently annotated in the in-house library using their spectral data and retention indices. Unidentified metabolites lacking matches in the in-house library were annotated based on their retention indices. Identified metabolites spanned various metabolic classes, such as amino acids, fatty acids, amino sugar derivatives, and organic acids. Significantly higher levels of glucuronic acid, glucose, and fructose were observed in cachectic patients compared to non-cachectic patients. Conversely, lower levels of erythronic acid, lysine, methionine, ornithine, homocysteine, threonine, alanine, proline, valine, leucine, tyrosine, 1,5-anhydro-d-glucitol, isoleucine, maltose, glutamine, and serine were noted in cachectic relative to non-cachectic patients (**Figure 3**). The metabolite heatmaps depicted distinct metabolic patterns between the cachectic and non-cachectic patients (**Figure 4**). Additionally, the volcano plot, visualizing statistical significance (p-value) versus magnitude of change (fold change), highlighted the altered metabolites within and between the cachectic and non-cachectic patients, with red denoting the up-regulated and blue indicating the down-regulated metabolites (**Figure 5**). Overall, these results provide robust evidence for a distinct metabolite profile associated with the clinical manifestation of cancer cachexia. Among significant metabolite alterations, the most noticeable ones are yet unknown altered serum levels of erythronic acid and glucuronic acid in human cancer cachexia (**Figures 3**-**5**). Moreover, the volcano scatterplot outlines that erythronic acid showed the highest magnitude of down-regulation and glucuronic acid the highest magnitude of up-regulation among the significantly changed metabolites between cachectic and non-cachectic patients (**Figure 5**).

### Pathway analysis highlights global metabolic changes in cancer cachexia

**Figure 6:**
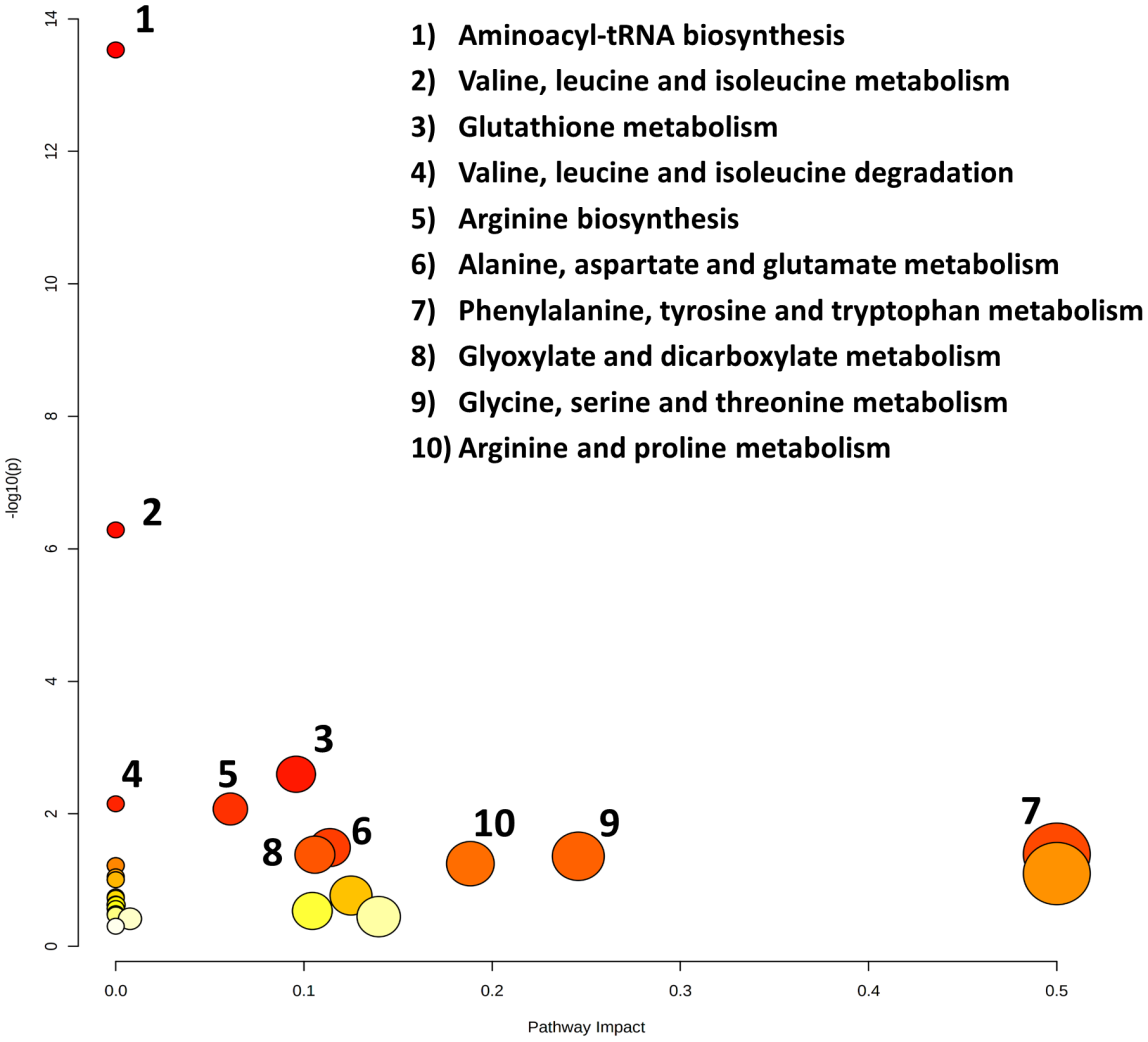
Topology map of altered metabolic pathways, which describes the impact of metabolites selected from a comparative *t*-test (p-value < 0.05), adjusted for multiple hypotheses testing by false discovery rate (FDR). The top ten altered metabolic pathways in the cancer cachexia group are 1. aminoacyl tRNA biosynthesis, 2. valine, leucine and isoleucine metabolism, 3. glutathione metabolism, 4. valine, leucine and isoleucine degradation, 5. arginine biosynthesis, 6. alanine, aspartate and glutamate metabolism, 7. phenylalanine, tyrosine, and tryptophan metabolism, 8. glyoxylate and dicarboxylate metabolism, 9. glycine, serine, and threonine metabolism, and 10. arginine and proline metabolism.

Noteworthy metabolic responses were observed when subjecting these significant metabolites to the pathway analysis tool (MetPA), revealing compelling insights into metabolic alterations within cachexia groups. Notably, the predominant pathways implicated in these responses included aminoacyl tRNA biosynthesis, valine, leucine, and isoleucine metabolism, glutathione metabolism, valine, leucine, and isoleucine degradation, arginine biosynthesis, alanine, aspartate, and glutamate metabolism, phenylalanine, tyrosine, and tryptophan metabolism, glyoxylate and dicarboxylate metabolism, glycine, serine, and threonine metabolism, and arginine and proline metabolism. A detailed topology map illustrating the impact of metabolites on these altered metabolic pathways is presented in **Figure 6**. This pathway map delineates the matched pathways based on their p-values from the pathway enrichment analysis and the pathway impact values from the pathway topology analysis. Overall, the integrative pathway analysis of the metabolite profile differences supports global metabolic changes associated with the manifestation of cachexia in cancer patients, predominantly affecting the amino acid (AA), protein, and glutathione metabolism.

### Robust logistic regression model predicts cancer cachexia with high accuracy

**Figure 7:**
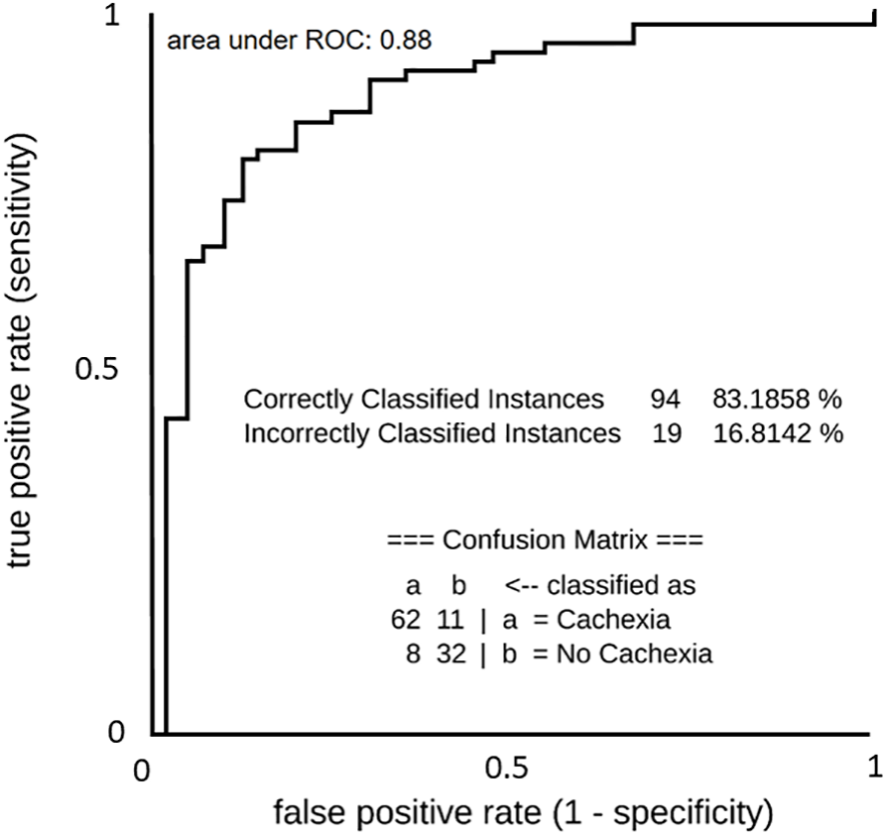
ROC curve of predictive machine learning (ML) models (Simple Logistics) for binominal discrimination between cachectic and non-cachectic state. List below ROC curve shows the confusion matrix and accuracy value of the model.

To account for potential combinatorial effects and interrelations among metabolites in our dataset, we implemented a purely prediction-oriented simple logistic ML-based model for binominal discrimination of cachexia states. Following the training of the simple logistic ML model using a 10-fold cross-validation strategy together with a meta classifier approach to make base predictors cost-sensitive, the predictive ML-based models achieved accuracy of 83.2% and an area under ROC value of 88.0% for the correct binominal discrimination of the samples according to patients cachexia state (**Figure 7**). Influencing predictors contributing to the correct binominal discrimination comprised 10 non-annotable and 5 annotable metabolites; the identifiable metabolites were erythronic acid, lactic acid, maltose, methionine, and ornithine (**Supplementary Table 2**). Despite of a small dataset and operations on subsamples through data splits (training/test data), the purely data-driven ML model yielded high predictive performance and identified erythronic acid as influencing predictor variable. This supports ML-based technologies as valuable tool for biomarker discovery, and indicates a benefit from taken account for combinatorial effects and interrelationships among metabolite alterations.

## Discussion

Cachexia alters the cancer patient’s metabolism with deleterious consequences, but the ability to understand and effectively treat cachexia remains an unmet need in cancer medicine (1–6). We pursued this by untargeted GC-MS-based metabolomics of overnight-fasting serum samples from previously untreated metastatic cancer patients presenting with and without cachexia according to validated diagnostic criteria agreed upon by international consensus (1–3). The cachectic patient group displayed considerable variations in 38 metabolites, of which 19 annotable metabolites belonged to metabolic classes such as amino acids, sugars, fatty acids, organic acids, and amino sugar derivatives. The significant differential 19 metabolites, mainly encompassing sugars and amino acids, distinguished accurately the cachectic state in both statistical and ML-based models. Pathway analysis found several pathways to which the metabolites contribute in several ways. To our best knowledge, this study is the first to report decreased erythronic acid and increased glucuronic acid levels in blood in human cancer cachexia. As discussed further, our work broadens the metabolic cancer cachexia landscape and may provide a resource for future study directions.

Elevated serum levels of glucose and fructose are the first noticeable metabolic feature of our cachectic cancer patients. Insulin resistance (IR) induced by the tumor upon the host is commonly present in human cancer cachexia (27, 28). Fasting hyperglycemia, as seen in our cachectic patients, also defines type 2 diabetes mellitus (T2DM) and is largely secondary to inadequate action of the major glucose-lowering hormone insulin (28). IR displays tissue-specific functional alterations in muscle, liver, and adipose tissue, which are unable to mount a coordinated glucose-lowering response, involving cellular uptake of glucose, suppression of gluconeogenesis and lipolysis, and glycogen synthesis (29). In cachectic cancer patients, the disturbed insulin-stimulated anabolic fluxes may shunt substrates to support the tumor anabolism. Tumors benefit from hyperglycemia, as they consume 200x more glucose than normal tissues to generate energy (30). This process termed anaerobic glycolysis (Warburg effect) produces large quantities of lactate, which can be converted back to glucose via gluconeogenesis in the tumor itself (31) and/or in the liver via the Cori cycle (32). These mechanisms, which maintain glucose supply but avoid lactate acidosis, may be present in our cachectic cancer patients, as they exhibited increased glucose but normal lactate serum levels. Notably, IR is sustained by low-grade inflammation in obesity (33), T2DM (34), and cancer cachexia (35), leading to some overlap in metabolic programming (36). However, clinical outcomes from testing the anti-T2DM drug metformin in anti-cancer therapy have been disappointing (37). Moreover, IR-related defects in obesity and T2DM are readily reversal by weight loss and hypocaloric nutrition, whereas weight loss persists in cancer cachexia despite hypercaloric nutrition. Therefore, future research might focus on investigating tumor-induced mechanisms that specifically sustain IR, glycemia, and fructose circulation in cancer cachexia. Notably, fructose has emerged as an important driver of both IR (38) and the Warburg effect in cancer cells (39). The increased serum fructose signal found in this study supports the inhibition of fructose-induced metabolic alterations as a reasonable approach to reverse cancer cachexia (40).

The second noticeable serum profile alteration in cachectic patients is the decrease of 13 circulating amino acids (AAs). As cancer cachexia refers to a state with increased muscle protein degradation and AA release into the blood circulation (41), this uniform trend likely reflects a disproportional high AA consumption. Tumors have high demands for AAs for energy production and nucleotide, lipid, and protein synthesis needed for tumor growth (42, 43). Especially glycine, serine, homocysteine, and methionine needed for one-carbon metabolism and glutamine needed for glutaminolysis are essential AAs to support tumor metabolism (44–46). The decline of these 5 AAs in our cachectic patients may point to increased one-carbon metabolism and glutaminolysis in cachexia-associated tumors. The three branched-chain AAs (BCAAs: isoleucine, leucine and valine) are carbon and nitrogen suppliers for energy demands and protein synthesis and sustain the TCA cycle and lipogenesis by providing acetyl-CoA, which is essential for histone acetylation and epigenetic modification (47, 48). BCAA serum levels are increased in IR and T2DM but decreased in several critical illnesses suggesting that the lower cachexia-associated BCAA levels may reflect increased supply to both tumoral and non-tumoral sites (e.g., immune cells sustaining inflammation, hepatic gluconeogenesis) (49, 50). The biological roles of the decreased AAs ornithine, proline, tyrosine, lysine, and alanine are multifaceted. Ornithine helps convert toxic ammonia to urea by the urea cycle and is a key substrate for excess polyamine production in many cancers (51, 52). Proline is involved in collagen and polyamine synthesis, tissue repair, and redox reactions (53, 54). Tyrosine is a precursor of neurotransmitters and a receiver of phosphate groups by way of protein kinases in signal transduction and regulation of enzymatic activity (55). Lysine plays roles in protein synthesis and structure, cross-linking of collagen polypeptides, histone modification, immune response, and tissue repair (56–58). Alanine, released into the bloodstream from muscle proteolysis, serves as major AA for protein resynthesis but also drives the glucose-alanine cycle (Cahill cycle), which regenerates glucose from alanine via hepatic gluconeogenesis (59, 60). Notably, we found lower serum signals in 11 of the 20 proteinogenic AAs (non-proteinogenic: homocysteine, ornithine), of whom 10 (all but lysine) could also act as substrates for gluconeogenesis. Despite the differences in the metabolism of individual AAs, the simultaneous decrease of AAs likely reflects alterations in tumor-associated cachexia-causing pathways that simultaneously affect all of them.

Altogether, cachectic patients exhibited 3 significantly elevated (including glucose and fructose) and 16 decreased (mostly AAs) circulating metabolites. The significance of the distinct metabolite profile for distinguishing cachexia states is shown by the PLS-DA model, which yielded distinct clusters with 85.6% accuracy, and supported by ML-based models, which identified a metabolic signature achieving 83.2% accuracy and an area under the ROC value of 88.0%. Pathway analysis of the observed metabolite variations indicated 10 metabolic pathways to be most significantly involved in cancer cachexia. Among these, the significance of aminoacyl-tRNA biosynthesis supports the crucial role of protein biosynthesis (61, 62), whereas the affected glutathione metabolism outlines the importance of detoxifying and antioxidant processes (63, 64). Collectively, the 7 AA-related metabolic pathways suggest that cachexia-associated tumors display a dependency on AA metabolism. In line with the AA shortage, one may consider AA deprivation to limit tumor anabolism or AA supplementation to limit body catabolism. However, in the tumor-bearing cachectic state, tumor anabolism overrides host catabolism, and AA deprivation may rather exacerbate cachexia and AA supplementation promote tumor growth (65). Instead, targeting the metabolic rewiring behind distinct metabolite dependencies of cachexia-associated tumors, but not normal tissues, may counteract both cancer and cachexia. Metabolic reprogramming, a key distinguishing cancer hallmark, includes unique demands for glucose, fructose and AAs to fuel critical pathways needed for energy, biosynthetic, methylation, acetylation and reductive metabolism to support tumor growth (28, 39, 66, 67). Commonly affected components of metabolic reprogramming include the one-carbon metabolism encompassing the folate and methionine cycles (43, 44), glutaminolysis (45), anaerobic glycolysis (30), glutathione metabolism (63, 64), the pentose phosphate pathway (68), polyamine synthesis (69), extracellular matrix (ECM) modelling (70), and protein glycolisation (71). Despite the influence of the gut microbiome, diet, and genetics on the human blood metabolome (72), many of the serum metabolite changes seen in our cancer cachexia cohort hint towards a biochemical foundation in overactivated cancer metabolism pathways in cachexia-associated tumors. Understanding cancer cachexia as a cancer metabolism syndrome would imply several novel means for pharmaceutical intervention against cancer, and, by reducing the catabolic drive, against cachexia (28, 39, 46, 62–74).

To our best knowledge, this study is the first to demonstrate a link between decreased erythronic acid levels in blood and cancer cachexia. The consistency of our FDR-corrected statistical tests (highest downregulated metabolite) and ML-based analyses (highest classifier importance) support the biological relevance of this unanticipated finding. However, the factors influencing circulating levels of erythronic acid are poorly characterized. Elevated serum levels of erythronic acid have been found in patients with transaldolase deficiency, which represents a defect in the non-oxidative branch of the reductive pentose-phosphate pathway (PPP) (75). Conversely, reduced erythronic serum levels may reflect increased PPP activity in cancer cells to generate 5-carbon sugars used in nucleotide, DNA and RNA synthesis and to supply reductive NADPH to counteract oxidative damage and support lipogenesis (68). The reduced levels of erythronic acid may be also a result of scavenging reactions against hydroxyl radicals in cachexic patients (76). Notably, erythronic acid is a by-product of the degradation of N-acetylglucosamine (GlcNAc) caused by reactive oxygen species (ROS) (77). Protein GlcNAcylation is the most common posttranslational modification of proteins by sugars, which affects numerous cellular functions, including metabolic enzyme activities (78). Serum erythronic acid levels may reflect alterations of the synthesis and/or degradation of GlcNAc and/or protein GlcNAcAcylation, which impacts metabolic programming in cancer, including the direction of glucose into the PPP to support tumor growth (79). However, further studies are required to assess whether these hypothetical or other mechanisms influence circulating levels of erythronic acid. Isotope-assisted metabolomics approaches may be a starting point to explore the currently unknown metabolism and turnover of erythronic acid in cancer cachexia.

In the context of cachexia, to our knowledge, this study is also the first to report increased levels of glucuronic acid in the blood. Notably, population-based studies outline glucuronic acid as a biomarker of all-cause mortality and healthspan-related outcomes (80). Several mechanisms may contribute to circulating levels of the glucose metabolite glucuronic acid, which participates in detoxification processes and ECM modelling (81, 82). Firstly, decreased glycosyltransferase activities, as seen in hepatic dysfunction, lead to decreased toxin-conjugation and, hence, increased glucuronic acid and toxin levels in the bloodstream (83). Secondly, cleavage of glucuronide toxin-conjugates by gut microbial ß-glucuronidases can counteract glucuronidation and hepatic-enteric detoxification and make deconjugated glucuronic acid and toxins available for reabsorption into the bloodstream (enteric-hepatic recycling) (84). Thirdly, human ß-glucuronidase, which localizes primarily in lysosomes, leads to hydrolytic liberation of glucuronic acid during the remodeling of the ECM (85). Low-grade inflammation, a typical feature accompanying cancer cachexia, can amplify the release of human ß-glucuronidase into the bloodstream, where it cleaves glucuronidated conjugates and contributes to circulating glucuronic acid and toxin levels (80, 82, 86). In most clinical scenarios, elevated glucuronic acid levels are likely the result of increased ß-glucuronidase activity, ECM remodeling, inflammation and/or cell death by concurrent disease (80–86). Recent drug developments emphasize ECM normalization and ß-glucuronidase inhibition as novel strategies in anti-cancer treatment (70, 87), which could also favorably affect the body´s glucuronic acid, toxin-conjugation, and detoxification metabolism. To bring the potential anti-cachexia effects of these anti-cancer drugs into perspective, clinical trial designs may expand patient-centered efficacy endpoints toward clinical benefits on symptoms and quality of life to cancer patients suffering from cachexia (88).

For opening up new diagnostic and therapeutic options for cancer cachexia, global metabolic changes and combinatorial effects within metabolomics data are of particular interest. Pathway analysis revealed protein and glutathione metabolism to be involved in cancer cachexia. This correlates with previous work supporting sarcopenia as important feature of cancer cachexia, including a deranged protein metabolism, likely caused by mitochondrial dysfunction in cachectic skeletal muscle tissue (1, 4, 65, 89). Disturbance in glutathione metabolism, the most important detoxifying antioxidant system in human tissues, has been shown to be implicated in increased resistance and toxicity to anti-cancer therapy in cachectic cancer patients (1, 4, 63, 64). Remarkably, purely data-driven ML models, taking into account the combinatorial effects of altered metabolites, yielded high performance for prediction of the cachexia state. The metabolites contributing to ML-based prediction, namely erythronic acid, lactic acid, maltose, methionine and ornithine, reveal intriguing metabolic adaptations. Erythronic acid´s levels may be linked to detoxification mechanisms involving GlcNAc and protein GlcAcylation (76–78) and PPP activity (79). Lactic acid correlates well with long-recognized resting energy expenditure in cancer cachexia, which has been related to futile metabolic cycling including an overactivation of the Cori cycle (7, 31, 32). Ornithine and methionine contribute to the urea cycle, glutathione synthesis with interconnection to one-carbon metabolism, and polyamine production (44–46, 69, 90). Research on the relationship between maltose and cancer cachexia is limited, necessitating further investigation to establish potential connections. Overall, contrasted to previous work in cancer cachexia research, we found well-known (glucose, AAs), less-recognized, but potentially important (fructose, maltose) and yet unknown (erythronic acid, glucuronic acid) metabolite alterations. The metabolite profile as a whole points to global metabolic changes related to cancer cachexia, which are in part long-recognized (e.g. altered glucose, protein, and glutathione metabolism, IR-like state, activated Cori cycle), less-recognized but potentially important (e.g., fructose and polyamine metabolism), or yet unknown (e.g. PPP activation, GlcNAcAcylation, reduced glucuronidation-based detoxification). The robust predictive performance of ML-based models may impact on future research directions and research methodologies. These findings point to a stronger focus on combinatorial effects of metabolic changes that collectively contribute to the development of cancer cachexia. Further, they support ML technologies as valuable tool for biomarker discovery. The validation of a common serum metabolite biomarker panel for early detection of cancer cachexia would provide tremendous advance in the design of clinical trials for new preventive and/or therapeutic interventions (91).

Our study has limitations. First, we used BMI-adjusted WL as the agreed and validated main diagnostic criterium of the internal consensus-definition for cancer cachexia (1–3). The legitimacy of applying this diagnostic criterium in our study cohort is supported by several items representing other cachexia domains that are easily applicable in clinical practice. However, future studies may benefit from additional muscle mass measurements to better assess the role of sarcopenia. Second, the case-control design using cancer patients without cachexia as control isolates results to cachexia as opposed to other phenomena associated with cancer. However, future research may benefit from the inclusion of healthy controls to examine how cancer affects early stages in the cachexia trajectory, which could guide clinical trial designs focusing on prevention, rather than treatment, of cachexia. Third, the serum metabolite profile was assessed only once. This does not represent intra- and inter-day variation of metabolites, which can confound signal detection in metabolomics research. Fourth, covariables, such as diet, medication, diabetes, patient-related factors and cancer type, may affect levels of serum metabolites. There was almost equal distribution of covariables without significant difference between the two patient groups analyzed. Further, pooling samples minimized inter-individual variation, making substantive findings easier to find (92). However, covariables were not assessed in this cross-sectional single-center study by downstream analysis to account for confounding effects. The overall small sample size, the small subgroup sizes but high numbers of metabolite features per sample, and the design-based constraints to measure effect sizes of intra- and interindividual variation in our dataset limited us to produce meaningful data in this respect. Further larger-scale, multi-centric, and longitudinal designed studies in independent patient populations, that include substantially increased numbers for different cancer types and pay attention to intra- and inter-day variation of serum metabolites, are needed to explore confounding factors, variability of metabolite levels, trends of metabolite level changes related to cancer cachexia progression over time, and to verify the extent to which the findings presented here are generalizable. Fifth, metabolome analyses of body fluids are challenging. To obtain high-quality samples and reproducible results, we applied a strict work-up according to recently published guidelines (22) for sample collection, metabolite extraction, quality control, GC-MS measurement, and data acquisition. Further, we applied strict statistical and ML-based (e.g., 10-fold cross-validation, cost-sensitive base classifier) methods to control overfitting, false discovery, and data misinterpretation. The consistent results obtained by significance-based and prediction-oriented ML-based analyses lend strengths to our findings. Finally, untargeted metabolomics is limited by the ability to identify unknown metabolites. However, no metabolomics approach can be completely comprehensive, and our identified metabolite cluster has biological plausibility. However, despite the aforementioned study limitations, we believe that our dataset and especially our new findings may contribute to the literature, may provide a resource for comparisons across patient cohorts with cancer cachexia and/or other metabolic diseases, and could stimulate future investigations in the field.

## Conclusions

In conclusion, we newly describe altered serum levels of erythronic acid and glucuronic acid as a characteristic feature of cancer cachexia, potentially linked to intra-tumoral PPP activation and impaired body detoxification. Further, we found a distinct serum metabolite profile of cancer cachexia, with glucose, fructose and AAs being the most disturbed metabolites. Some serum metabolite alterations could reflect the supply of overactive metabolic pathways in cachexia-associated tumors needed for energy, biosynthetic, epigenetic and reductive metabolism. Additional studies connecting measurements from both tumor and body metabolism may be an interesting direction to identify actionable targets for distinct metabolic needs of cachexia-associated tumors, but not normal tissues. Altogether, our findings broaden the scope of metabolic vulnerabilities, dependencies and targets in cancer-associated cachexia that can help define testable hypotheses about mechanisms of action and/or design novel therapy approaches to improve patient outcomes in an important field of cancer patient care.

## Data availability statement

The raw data supporting the conclusions of this article will be made available by the authors, without undue reservation.

## Ethics statement

The studies involving humans were approved by Aerztekammer Hamburg, Protocol number: V5649, Date: 23.10.2017. The studies were conducted in accordance with the local legislation and institutional requirements. The participants provided their written informed consent to participate in this study.

## Author contributions

TM: Data curation, Formal Analysis, Investigation, Supervision, Writing – original draft, Methodology, Software, Validation, Visualization. KH: Formal Analysis, Methodology, Data curation, Funding acquisition, Investigation, Supervision, Validation, Writing – review & editing. MS: Conceptualization, Data curation, Formal Analysis, Methodology, Software, Supervision, Visualization, Writing – review & editing. TI: Data curation, Formal Analysis, Supervision, Validation, Writing – review & editing. RS: Conceptualization, Data curation, Investigation, Supervision, Validation, Writing – review & editing. RG: Data curation, Formal Analysis, Methodology, Software, Visualization, Writing – review & editing. TV: Data curation, Formal Analysis, Supervision, Validation, Writing – review & editing. HW: Data curation, Formal Analysis, Investigation, Supervision, Validation, Writing – original draft. AS: Conceptualization, Data curation, Formal Analysis, Funding acquisition, Investigation, Project administration, Supervision, Writing – original draft.

## Supplementary material

Supplementary Figure 1 Principal component analysis score plot depicting clustering of cachectic (red) versus non-cachectic (green) cancer patients.
