# Supplementary figures and images for: Metabolomics analysis reveals novel serum metabolite alterations in cancer cachexia
Source: Front Oncol. 2024 Feb 20;14:1286896. doi: 10.3389/fonc.2024.1286896 (PMC10915872; doi:10.3389/fonc.2024.1286896)

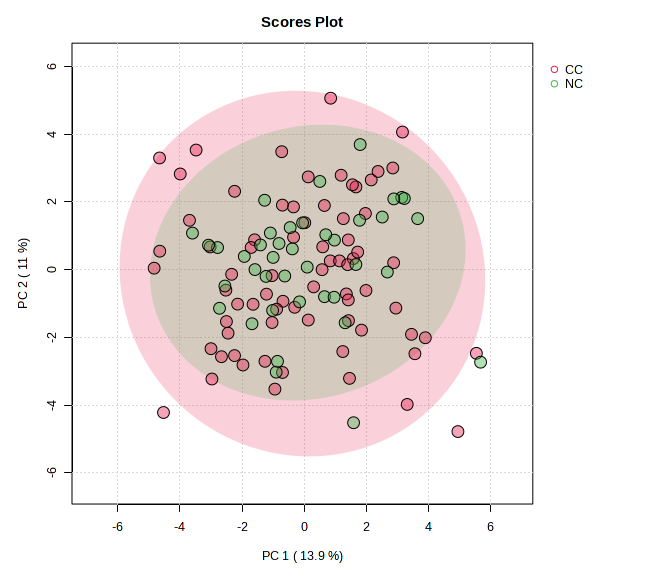

Supplement: Supplementary Figure 1 — Principal component analysis score plot depicting clustering of cachectic (red) versus non-cachectic (green) cancer patients. [file Image_1.tif]
