# Supplementary material for: Metabolomics analysis reveals novel serum metabolite alterations in cancer cachexia
Source: Front Oncol. 2024 Feb 20;14:1286896. doi: 10.3389/fonc.2024.1286896 (PMC10915872; doi:10.3389/fonc.2024.1286896)
Supplement: Supplementary file 3 [file Table_2.docx]

| Supplementary Table S2. Classifier composition and according coefficients in machine learning-based Simple Logistic models using serum metabolome data for classification between cachectic (n = 78) and non-cachectic (n = 42) cancer patients. | |
| --- | --- |
| Coefficient | Metabolite |
| -0.67335 | Erythronic acid |
| 0.56282 | Lactic acid |
| -0.49648 | Maltose MP |
| -0.32908 | Methionine |
| 0.41931 | RI1157.26 |
| -1.27951 | RI1551.98 |
| -1.06825 | RI1611.90 |
| 1.54837 | RI1682.99 |
| -0.23481 | RI1861.25 |
| -0.47374 | RI2058.22 |
| -0.18863 | RI2166.59 |
| 0.12883 | RI2421.11 |
| -0.27547 | RI2960.14 |
| -0.53844 | RI3593.15 |
| -0.50989 | Ornithine |
